# Optimal combination of microcoils, flow control, and n-butyl cyanoacrylate–Lipiodol–iopamidol (2:3:1) for feasible embolization of medium-sized arteries in an in vitro vascular model

**DOI:** 10.1007/s11604-024-01667-4

**Published:** 2024-09-28

**Authors:** Takeshi Suzuki, Jun Matsuda, Yoshinori Tsukahara, Ayumi Ohya, Akira Yamada, Masahiro Kurozumi, Yasunari Fujinaga

**Affiliations:** Department of Radiology, Shinshu University School of Medicine, 3-1-1 Asahi, Matsumoto, Nagano, 390-8621 Japan

**Keywords:** N-butyl cyanoacrylate–Lipiodol–iopamidol, Microcoils, Flow control, Embolization, Medium-sized arteries

## Abstract

**Purpose:**

To evaluate the behavior of n-butyl cyanoacrylate–Lipiodol–iopamidol at a ratio of 2:3:1 (NLI231) with and without microcoils and/or flow control in embolization of medium-sized arteries in an in vitro vascular model.

**Materials and methods:**

A vessel model representing a common hepatic artery was prepared. Six scenarios were set for embolization, each ran three times: 1) NLI231 injected alone with flow control to 0 ml/min during and up to 5 min after embolization; 2) NLI231 injected into a mesh of microcoil of 5% density with the flow control; 3) NLI231 injected into a microcoil of 10% density with the flow control; 4) NLI231 injected alone without the flow control; 5) NLI231 injected into microcoil of 5% density without the flow control; 6) NLI231 injected into a microcoil of 10% density without the flow control. The microcoils were delivered to the embolization site, and NLI231 was injected. After 1 h of observation, distal filters were collected, and grades of migration (I = none, II = partial, III = almost all–all) were assessed for each scenario.

**Results:**

Embolization was achieved in scenarios with NLI231 and microcoils regardless of flow control (*p* < 0.01). NLI231 did not migrate in scenarios with microcoils and flow control (*p* < 0.05). NLI231 with microcoils without flow control can embolize the vessel, but partial migration occurred, and the distal distance of the NLI231 complex from the embolization site was longer (*p* < 0.01).

**Conclusion:**

Combining sparse coiling with NLI231 may be feasible but is limited to use when flow control is available, or where distal embolization is permissible to some extent.

## Introduction

There are often cases requiring urgent, wide-ranging coil embolization for active bleeding associated with damaged medium-sized arteries. Examples include patients with extravasation or pseudoaneurysms onto the common hepatic artery after pancreaticoduodenectomy or the splenic artery following severe pancreatitis induced by leakage of pancreatic enzymes. However, a sufficient quantity and variety of microcoils are often lacking in angiographic suites to effectively embolize the target arteries. Immediate delivery from retail sellers to the hospital is not always available. Furthermore, even institutions that have enough coils should need cost savings. Additionally, in situations where coagulation has failed, such as hemorrhagic shock and disseminated intravascular coagulation, embolic support by autologous thrombus formation cannot be expected, making hemostasis difficult to achieve with a sparse coil density.

Liquid embolic materials such as n-butyl cyanoacrylate (NBCA) have become widely used in emergency arterial embolization and are mixed with other substances like Lipiodol in various proportions to adjust the visibility and speed of embolization [1, 2]. N-butyl cyanoacrylate–Lipiodol–iopamidol (NLI) is one of the safer combinations of liquid embolic material. Since it has high visibility and viscosity, it is visible under fluoroscopy and does not adhere easily to catheters [3–6]. Fukuda et al. [4] reported that NLI at ratios of 2:3:1 (NLI231) and 1:4:1 (NLI141) are feasible ratios in practice, and among these, NLI231 has a higher viscosity and lower tendency to migrate compared to NLI141. Therefore, we hypothesized that NLI231 would be more likely to entangle with microcoils during embolization than NLI141. Fukamatsu et al. [7] reported that combining sparse coiling with NLI231 allows for speedy embolization of a large aneurysm. Higashino et al. [8] reported balloon-assisted arterial embolization of pseudoaneurysms using NLI231. However, it is still unclear whether microcoils and flow control are required for feasible embolization of medium-sized arteries with NLI231. The purpose of this study is to evaluate the behavior of NLI231 with and without microcoils and/or flow control in embolization of medium-sized arteries in an in vitro vascular model.

## Materials and methods

### In vitro vascular model

**Fig. 1:**
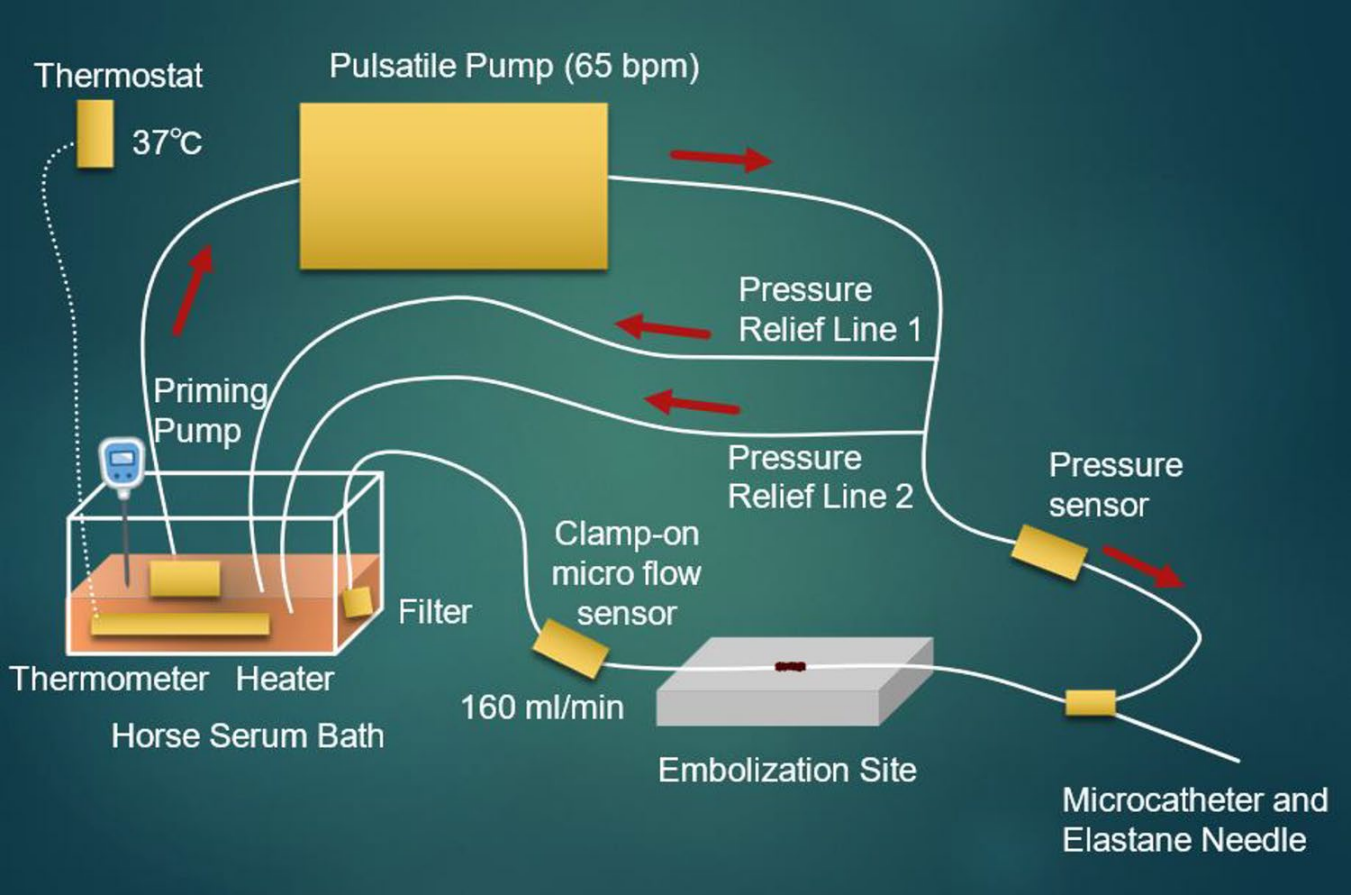
In vitro vascular model. Arrow: flow direction. A hydrophilic polymer-coated vessel model resembling a common hepatic artery was prepared using polyurethane resin tubing coated with vinyl ether maleic anhydride. Donor horse serum was circulated through the vessel model using priming and pulsatile pumps, maintaining a flow rate of approximately 160 ml/min at a temperature of 37 °C. To prevent excessive pressure buildup during embolization, two pressure relief lines were connected from the proximal side of the embolization site to the bath

A hydrophilic polymer-coated vessel model representing a common hepatic artery [(inner/outer diameter 5/7 mm, polyurethane resin tubing with lumen coated with vinyl ether maleic anhydride (Hagitec, Yotsukaido, Japan)] was prepared (Fig. 1) by referring to previously described experiments [9–11]. Donor horse serum (S-DHS-EU-015, Serana, Pessin, Germany) was circulated by plastic tubes placed in the bath and connected to priming and pulsatile pumps (Kyoto Kagaku, Kyoto, Japan). We set a flow rate of the embolization site at approximately 160 ml/min (65 bpm) based on the assumed total liver flow rate in the past studies [12, 13], and the temperature of the serum was kept at 37℃ by a thermostat, a thermometer, and a heater in the bath. We ran two pressure relief lines from the proximal side of the embolization site to the bath as collaterals to prevent extreme elevation of the pressure in the tube during successful embolization. A hemostasis valve (Passage™, Merit Medical, South Jordan, UT, USA) was placed just proximally to the embolization site, and we inserted catheters and needles through it. A pressure sensor (Meritrans DTXPlus®, Merit Medical, South Jordan, UT, USA) was placed directly proximally to the valve. We placed a clamp-on micro flow sensor (FD-XC8R2/FD-XS8E, Keyence, Osaka, Japan) distal to the embolization site. We placed a filter made of a piece of gauze and a rubber band at the distal end of the tube to collect migrated NLI231.

## Flow control

The flow rate was controlled by closing and opening the three-way stopcock connected to the proximal pressure sensor. After the mesh of microcoils was formed, the cock was closed immediately before the injection of NLI231, and the flow rate was set to 0 ml/min. The cock was then opened gradually over several seconds to fully open 5 min after injection of NLI231. The occlusion time was determined based on acceptable occlusion time without ischemia of vital organs that was suggested in past studies using balloon occlusion tests of the celiac artery [14] and internal carotid artery [15].

## Embolization scenarios

**Fig. 2:**
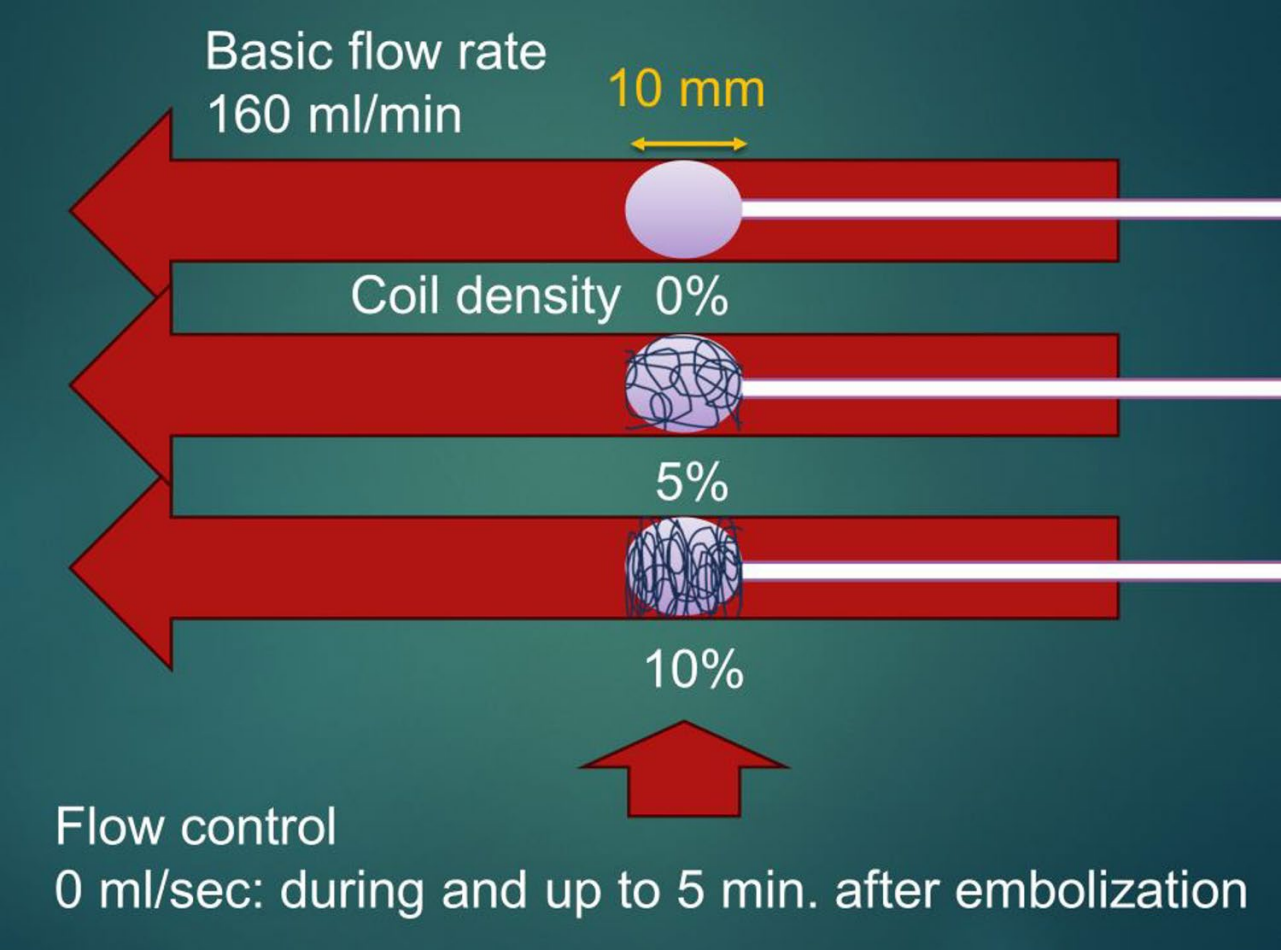
Embolization scenarios. The study conducted embolization using six different scenarios, each repeated three times. The scenarios involved injecting an NLI231 with or without flow control, into microcoil meshes of varying densities. The embolization range was set at 10 mm to calculate coil density and reduce costs. Embolization was considered successful when distal flow ceased

**Fig. 3:**
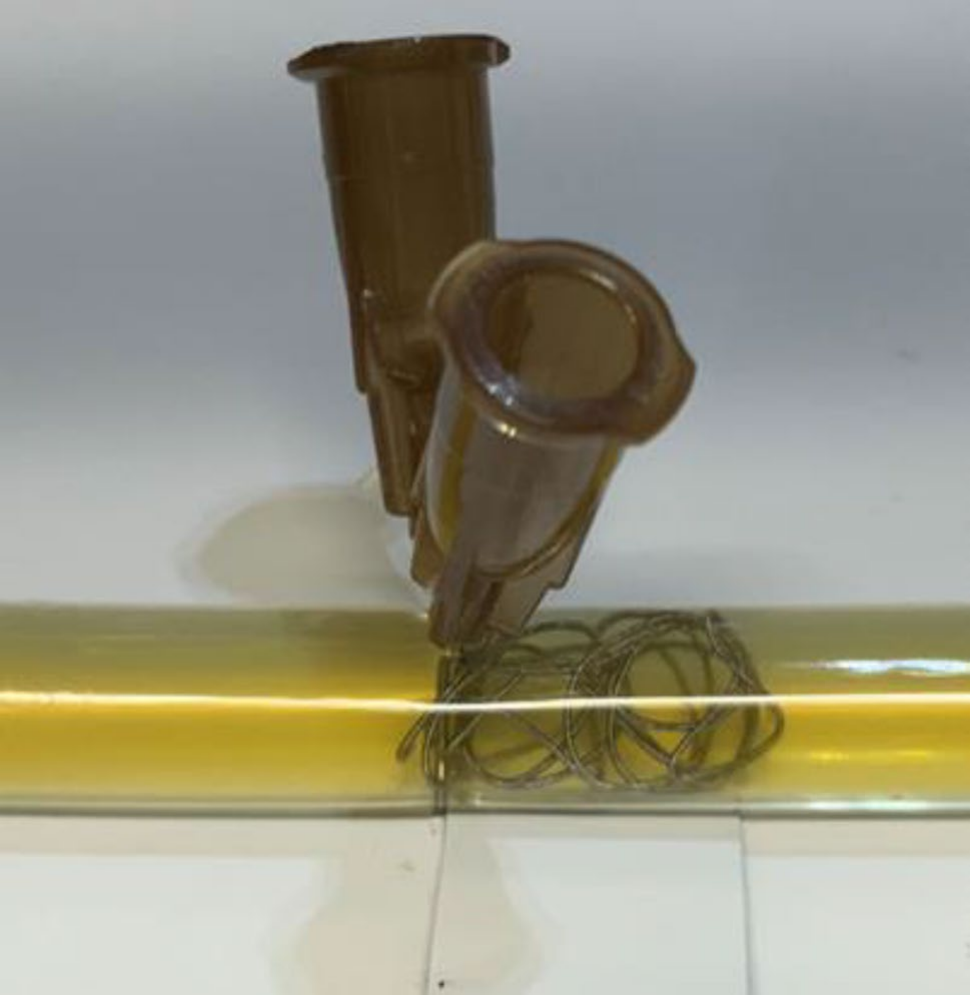
Embolization site. Microcoils were delivered using a microcatheter, and 26G needles were inserted as anchors at the embolization site. The coils were placed uniformly and detached

Six scenarios were set for embolization (Fig. 2): 1) we injected only NLI231 with flow control; 2) we injected NLI231 into a mesh of microcoil of 5% density (Target® 360°soft 6 mm 20 cm × 1, Striker, Fremont, CA, USA) with the flow control; 3) we injected NLI231 into a mesh of microcoil of 10% density (Target XL® helical 6 mm 20 cm × 1, Striker, Fremont, CA, USA) with the flow control; 4) we injected only NLI231 without flow control; 5) we injected NLI231 into a mesh of the microcoil of 5% density without flow control; 6) we injected NLI231 into a mesh of the microcoil of 10% density without flow control. We meticulously ran each scenario three times to ensure accuracy and reliability. These non-fibered microcoils were delivered to the embolization site with a microcatheter (1.7/2.4 F, Excelsior® SL-10®, Striker, Fremont, CA, USA). Before coiling, we inserted two needles transversely from opposite sides (26G, 13 mm, Nipro, Tokyo, Japan) at the distal end of the embolization site as an anchor (Fig. 3). The microcoils were placed so as to form as uniform a mesh as possible and were detached using InZone Detachment System (Striker, Fremont, CA, USA). We set the range of embolization at 10 mm so that we could calculate the coil density from the volume of the cylinder (base area x height) and to save the cost of the coils. Coil density was calculated with the following equation:

Coil density (%) = V_coil_/V_tube_ × 100,

V_coil_ = the volume of the coil = π (primary coil diameter /2) ^2^ × coil length,

V_tube_ = the volume of the tube = base area {π/4 × inner diameter (= 5 mm) ^2^} × length (= 10 mm).

**Fig. 4:**
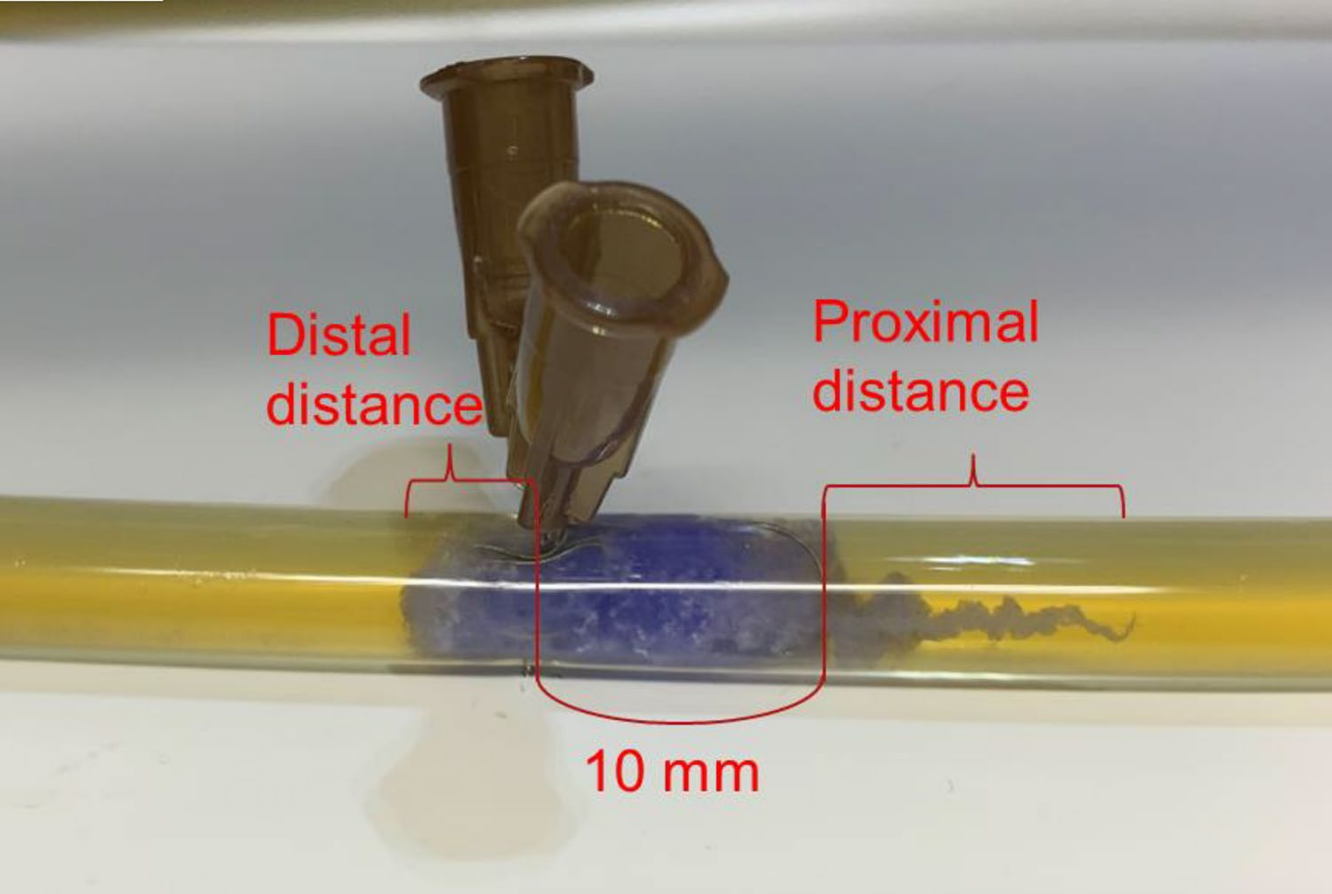
Measurement of distance. The distance of injected NLI231 from the target site was measured both proximally and distally

0.6 ml of NBCA　(Histoacryl®, B. Braun, Melsungen, Germany) was placed in a 2.5-ml locking syringe (Nipro, Tokyo, Japan) using a Cathelin needle (23G, 60 mm, Terumo, Tokyo, Japan), and 0.9 ml of Lipiodol (Lipiodol® Ultra Fluid, Guerbet, Aulnay-sous-Bois, France) was added. Then, 1.5 ml of the mixture of NBCA–Lipiodol, and 0.3 ml of 370 mg iopamidol/ml (Iopamidol「F」^®^, Fuji Pharma, Tokyo, Japan) was pumped in 15 times in 15 s using 2.5-ml locking syringes and a three-way stopcock (polyvinyl chloride free, L1-FL-LP, Top, Tokyo, Japan). Five minutes after the creation of NL231, 0.2 ml of the NLI231 was manually injected into the middle of the embolization site through the cannula of the 21G elastane needle (EV 21G × 200 mm, Hakko, Tokyo, Japan) using the 1-ml locking syringe (Nipro, Tokyo, Japan) immediately after injection of a small amount of 5% purified glucose (Otsuka Glucose Injection®, Tokyo, Japan). The cannula of the 21G elastane needle had a 0.028-inch inner diameter, and it was almost the same as that of high-flow microcatheters. Each run of the process was observed and recorded from the beginning of the embolization for 1 h, producing a 5-min real-time movie and a time-lapse movie of the remaining 55 min (iPad Pro ^®^, Apple, Cupertino, CA, USA). Finally, the distal filters were collected, and grades of migration (I = none, II = partial, III = almost all–all) were assessed for each scenario based on the gross visual inspection. The embolization was considered completed when the distal flow was 0 ml/min. We also measured the distance of the injected NLI231 from the target site proximally and distally (Fig. 4).

## Statistical analysis

We compared the grades of migration among the four groups (with microcoil with flow control; without microcoil with flow control; with microcoil without flow control; without microcoil without flow control) using the Steel–Dwass test and the success or failure of embolization using the Chi-squared test. We compared the range of the injected NLI231 from the target site between the two groups (with and without flow control) using the Mann–Whitney *U* test. *p* < 0.05 was considered to indicate significant differences.

## Results

**Fig. 5:**
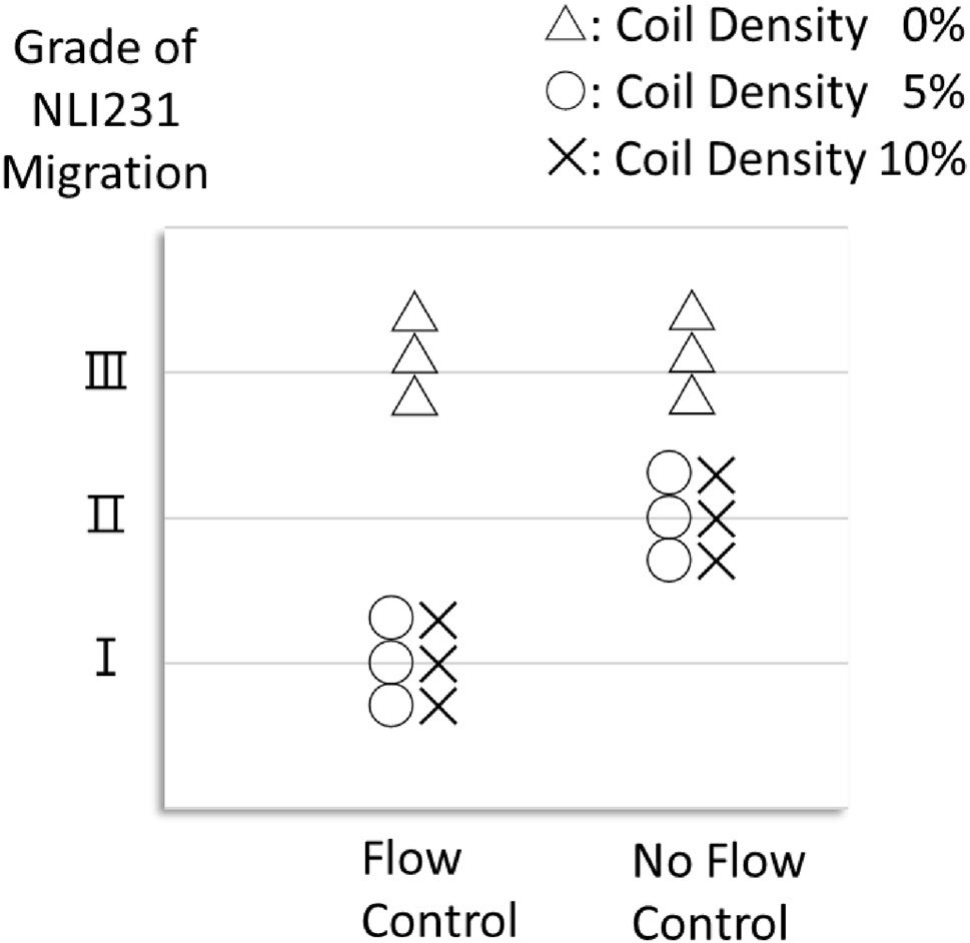
The degree of NLI231 migration. I = none, II= partial, III= almost all–all. There were no migrations in scenarios with all NLI231, microcoil, and flow control. Each plot reflects each of the three trials. Abbreviation: NLI231, n-butyl cyanoacrylate–Lipiodol–iopamidol at a ratio of 2:3:1

**Fig. 6:**
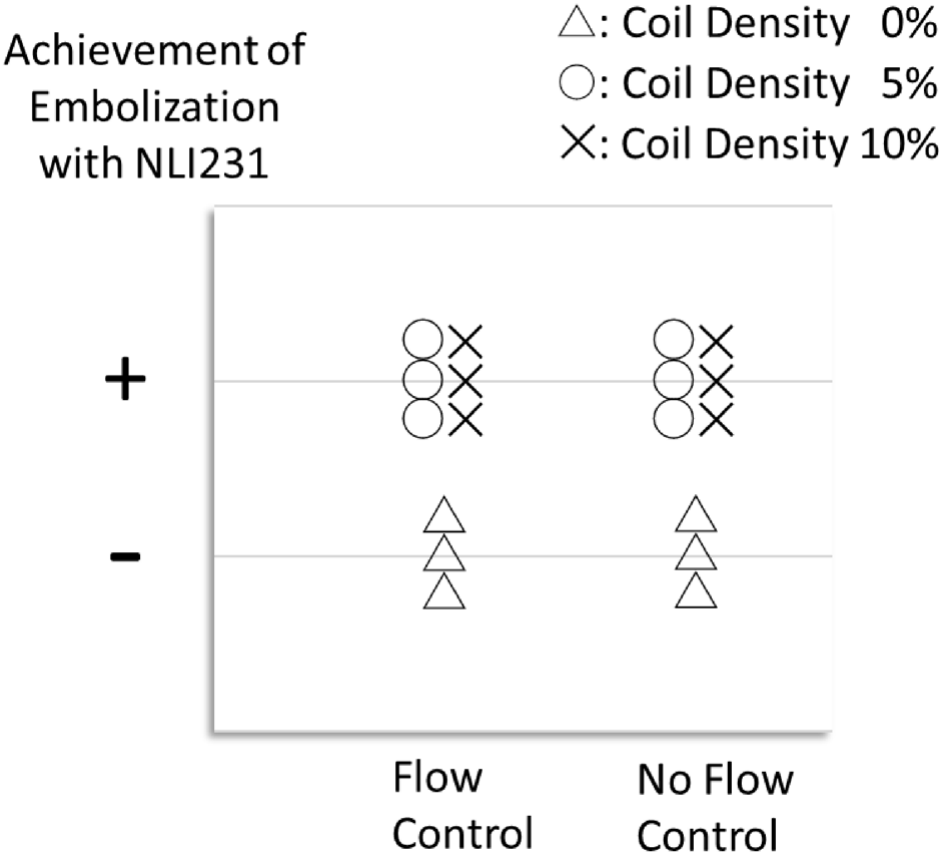
The achievement of embolization. +  = embolization was achieved (distal flow = 0 ml/min). − = embolization was not achieved (distal flow > 0 ml/min). Complete embolization was achieved in scenarios with NLI231 and microcoil regardless of the presence or absence of flow control. Each plot reflects each of the three trials. Abbreviation: NLI231, n-butyl cyanoacrylate–Lipiodol–iopamidol at a ratio of 2:3:1

**Fig. 7:**
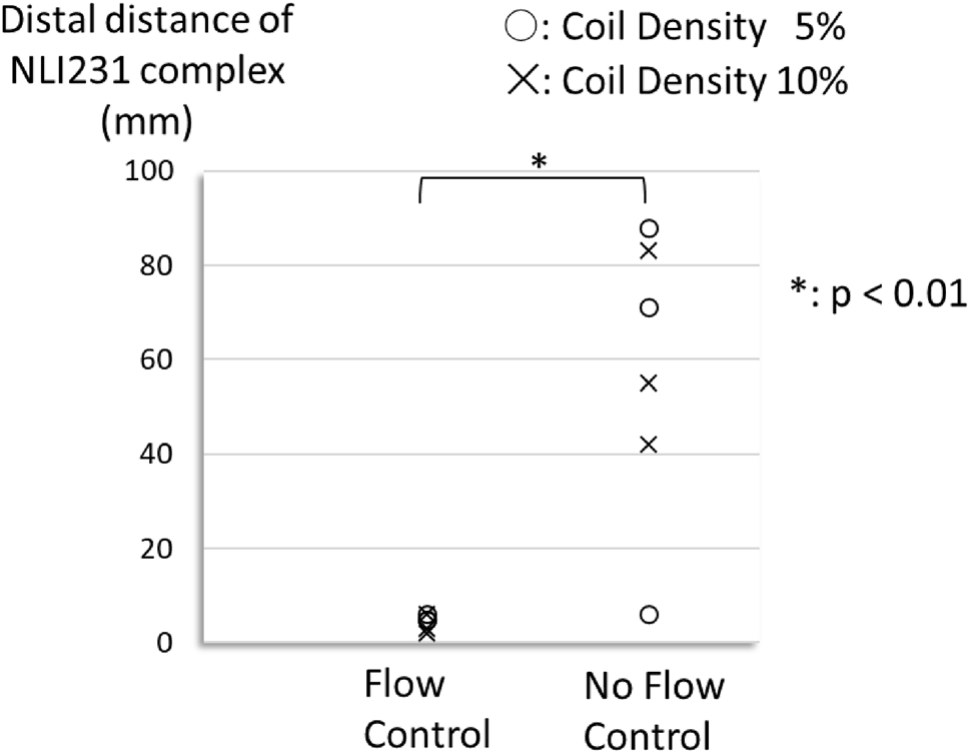
The distal distance of NLI231 complex at the embolization site. Distal distance (mm) of NLI231 complex at the embolization site with/without flow control. The distal distance of the NLI231 complex from the embolization site was longer without flow control than with it. The distance in coil density of 0% was unavailable because there was no NLI231 complex at the embolization site without microcoils. Each plot reflects each of the three trials. Abbreviation: NLI231, n-butyl cyanoacrylate–Lipiodol–Iopamidol at a ratio of 2:3:1

The main results are shown in Figs. 5, 6, 7. Complete embolization was achieved in scenarios with NLI231 and microcoil regardless of the presence or absence of flow control (Chi-squared test, *p* < 0.01). There were no migrations in scenarios with all NLI231, microcoil, and flow control (Steel–Dwass test, p < 0.05). NLI231 with microcoil without flow control can embolize the vessel, but partial migration occurred, and the distal distance of the NLI231 complex from the embolization site was longer: Median (interquartile range): 63 (33–84.25) mm vs. 5.5 (2.75–6) mm (Table 1); Mann–Whitney *U* test, *p* < 0.01 (Fig. 7). In scenarios with NLI231 alone with flow control, the NLI231 complex temporarily remained in the embolization site while flow control, but migrated at a mass after the flow restarted. In scenarios with complete embolization, the mean ± standard deviation systolic pressure on the proximal side of embolization was 151 ± 16.8 mmHg before embolization and 276 ± 16.9 mmHg after embolization. There was no adhesion of NLI231 to the cannula after injection in all.

**T﻿able 1:** The range of embolization

	Flow control			No flow control		
Trial	NLI231 coil 0%	NLI231 coil 5%	NLI231 coil 10%	NLI231 coil 0%	NLI231 coil 5%	NLI231 coil 10%
1	n/a	5/15	2/6	n/a	6/15	42/45
2	n/a	6/6	3/7	n/a	88/0	83/0
3	n/a	6/7	6/2	n/a	71/0	55/7

*NLI231*, n-butyl cyanoacrylate–Lipiodol–iopamidol at a ratio of 2:3:1

Distal/proximal distance (mm) of the NLI231 complex from the embolization site. n/a: not available because there is no NLI231 complex at the embolization site

## Discussion

Our in vitro vascular model revealed the behavior of NLI231 in combination with microcoils and with or without flow control. There were no migrations in scenarios with both microcoil and flow control. This suggests that NLI231 in combination with sparse coil placement and flow control may be feasible for embolization of medium-sized arteries. NLI231 and a mesh of sparse microcoil are both fragile alone. NLI231 is soft immediately after injection and migrates easily under fast flow conditions because it becomes harder slowly in the process of polymerization [4, 16]. Microcoil of 5 or 10% density is not enough to embolize the artery, considering the recommended density of 25% in the embolization of aneurysms [17]. However, similar to the relationship between cement concrete and reinforcement steel, the combination of NLI231 and microcoil with flow control led to successful embolization by trapping the NLI231 complex within a mesh of microcoil, acting as an anchor at the embolization site. Additionally, flow control prevented the migration of the NLI231 complex by inducing temporary stasis during the polymerization process, particularly during propagation from the surface toward the center of the NLI231 complex, as demonstrated by Li et al. [16].

Our results also suggest that microcoils with densities of both 5% and 10% are suitable as a scaffold for the NLI231 complex when flow control is applied and a distal anchor is present. Both densities are sparse, allowing the catheter tip to be easily visualized under fluoroscopy. Moreover, using these sparse microcoils can potentially reduce the cost and shorten the procedure time for extensive embolization.

NLI231 with microcoils without flow control can embolize the vessel, but partial migration occurred, and the distal distance of the NLI231 complex from the embolization site was longer. Under fast flow conditions without flow control, NLI231 migrates easily. While rapid injection of NLI231 into the embolization site may allow most of NLI231 to remain at the embolization site, it is impossible to prevent a partial migration. Therefore, the combined use of NLI231 and microcoils without flow control should be limited to cases where a certain degree of distal embolization is acceptable or where sufficient modification of distal blood flow is possible.

In scenarios that achieved embolization (distal flow = 0 ml/min), the systolic pressure on the proximal side of the embolization site was approximately 300 mmHg. This appears to be higher than the pressure observed in vivo, likely due to the presence of additional collaterals in actual vessels. Consequently, the proximal pressure would likely be even lower in vivo compared to our in vitro experimental setup.

There was no adhesion of NLI231 to the cannula after injection in any scenario, consistent with findings from previous studies [3–8]. Therefore, balloon catheters can be used for flow control without concern for adhesion. Non-adhesive liquid embolic materials such as LAVA ^®^ [18] and Obsidio ^®^ [19] may be used instead of NLI231, but they are expensive and not widely available.

The glue-in-plug technique has been reported by Ikoma et al. [5]. The glue-in-plug technique is superior to the microcoil–NLI231 combination in that it does not require an anchor and allows embolization over a short distance. However, the glue-in-plug technique cannot be used in cases where the vascular plug cannot be delivered to the target site due to strong tortuosity, or when the diameter of the vessel varies widely with extensive vascular injury. In these cases, the combination of NLI231 and microcoils would be a better option. While the plug has a fine and uniform mesh on its envelope, there are vacant areas where the NLI231 does not touch the mesh in the core. On the other hand, microcoils have an uneven overall gap; unless the center is very sparse, it may tangle with NLI231 from the beginning of injection, and it is unclear as to which is effective for the embolization with NLI231. It appears that this weak point of the plug could be compensated for by injecting the NLI231 after the microcoil is sparsely placed in the core of the plug, but further study is needed.

This study had several limitations. Although we simulated conditions similar to those in vivo, such as temperature, serum composition, and the hydrophilic surface of the vessels, which are important for the polymerization of n-butyl cyanoacrylate as demonstrated by Wang et al. [20], factors such as blood coagulation, arterial elasticity, blood viscosity, and variety of blood velocity were not taken into account. In particular, we used donor horse serum instead of human blood. This choice may result in slower polymerization and increased migration compared to a clinical setting. The absence of various factors, including the effects of clot formation that aid in coagulation and polymerization, contributes to these differences [21]. Therefore, further in vivo studies using blood and actual vessels are needed. Additionally, the number of trials was small, and variations in coil specifications, such as stock wire/primary/secondary diameter, shape, and material, could not be adequately assessed due to budget limitations. In this study, needles were used to anchor the embolized coils, assuming that the coil did not move with the flow. More coils will likely be needed in clinical practice to prevent coil migration than in this experiment. Finally, the long-term outcomes of the embolization procedures remain unknown.

## Conclusion

In embolization of medium-sized arteries, combining sparse coiling with NLI231 may be feasible, but is limited to use when flow control is available, or where distal embolization is permissible to some extent.
